# Intestinal Cell Barrier Function *In Vitro* Is Severely Compromised by Keratin 8 and 18 Mutations Identified in Patients with Inflammatory Bowel Disease

**DOI:** 10.1371/journal.pone.0099398

**Published:** 2014-06-10

**Authors:** Tina Zupancic, Jure Stojan, Ellen Birgitte Lane, Radovan Komel, Apolonija Bedina-Zavec, Mirjana Liovic

**Affiliations:** 1 National Institute of Chemistry, Ljubljana, Slovenia; 2 Medical Centre for Molecular Biology, Faculty of Medicine, University of Ljubljana, Ljubljana, Slovenia; 3 Institute of Medical Biology, Immunos, Singapore; Charité, Campus Benjamin Franklin, Germany

## Abstract

Keratin 8 and 18 (K8/K18) mutations have been implicated in the aetiology of certain pathogenic processes of the liver and pancreas. While some K8 mutations (K8 G62C, K8 K464N) are also presumed susceptibility factors for inflammatory bowel disease (IBD), the only K18 mutation (K18 S230T) discovered so far in an IBD patient is thought to be a polymorphism. The aim of our study was to demonstrate that these mutations might also directly affect intestinal cell barrier function. Cell monolayers of genetically engineered human colonocytes expressing these mutations were tested for permeability, growth rate and resistance to heat-stress. We also calculated the change in dissociation constant (K_d_, measure of affinity) each of these mutations introduces into the keratin protein, and present the first model of a keratin dimer L12 region with *in silico* clues to how the K18 S230T mutation may affect keratin function. Physiologically, these mutations cause up to 30% increase in paracellular permeability *in vitro*. Heat-stress induces little keratin clumping but instead cell monolayers peel off the surface suggesting a problem with cell junctions. K18 S230T has pronounced pathological effects *in vitro* marked by high K_d_, low growth rate and increased permeability. The latter may be due to the altered distribution of tight junction components claudin-4 and ZO-1. This is the first time intestinal cells have been suggested also functionally impaired by K8/K18 mutations. Although an *in vitro* colonocyte model system does not completely mimic the epithelial lining of the intestine, nevertheless the data suggest that K8/K18 mutations may be also able to produce a phenotype *in vivo*.

## Introduction

In the last 20 years intermediate filaments (IF) have been recognized as an important component of the cytoskeleton filament system and to have a variety of functions, far beyond the initial simplistic view of a mechanical support to cells. IF proteins have been shown to contribute to the overall cell architecture, migration, cell cycle and proliferation [1]–[4], and to have a role in cell signalling that can even affect target gene expression [5]–[8]. IF proteins are also being used in diagnostics as markers of distinct physiological changes in tissues during tumorigenesis [9], [10]. In addition, 93 pathological conditions have been associated with some 700 mutations in 73 IF proteins. The amazing progress made on IF protein biology started in the early 90's, when the first keratin gene mutations were identified and linked to a group of skin blistering disorders [11], [12]. Keratins are the largest family of IF proteins and are specific to epithelial tissues. The vast majority of the discovered several hundred keratin gene mutations are linked to hereditary disorders of the skin and skin appendages (nail, hair, teeth). However a number of mutations have been also identified in the simple epithelia keratins K8, K18 and K19 [13]. These are associated with pathologies of the liver, pancreatitis and inflammatory bowel disease (IBD). On the contrary to epidermal keratins, which when mutated mostly have a dominant-negative effect, the simple epithelia keratins appear to act as a susceptibility factor.

Crohn's disease and ulcerative colitis are the major types of IBD. Both are complex and chronic inflammatory conditions of the gastrointestinal tract, which are considered a combination of an altered immune response to luminal microbial flora, genetic factors (over 150 associated loci), and the effects of life style, diet and environment [14], [15]. The main symptoms are diarrhoea, abdominal pain, intolerance to certain types of food and fever. Both diseases are characterized by frequent ulcerations of the intestinal epithelium, which are considered important for the pathogenesis of IBD by causing altered barrier function and further exacerbating the immune response to toxins and pathogens normally present in the gut [16]. Therefore, intestinal fragility and altered barrier function are as important to the aetiology of IBD, as epidermal fragility and perturbed barrier function are to a large number of keratin-related skin disorders [17], [18].

Several years ago we published that severe K5/K14 gene mutations, which are associated with a severe skin blistering phenotype in patients with epidermolysis bullosa simplex, cause a down-regulation of cell junction proteins [19]. Since then several other reports indicated that keratin IFs are necessary for proper targeting and assembly of cell junction complexes at the cell membrane [20]–[22]. While this helps explain the fragility and diminished epidermal barrier function of a variety of genetic skin conditions, it also reminds us of some clinical features of IBD. We now know that a mutation in a cytoskeletal protein may affect its function on different levels, and not only affect protein structure *per se*. Although a mutation may not alter the structure of a protein to affect its primary function, it may still hinder the binding of associated proteins or interfere its interaction with other cytoskeletal components, as well as affect post-translational modification by obstructing site-specific changes like phosphorylation, glycosylation, acetylation and sumoylation. This may well be the case for mutations in simple epithelia keratins [23]–[25].

In this study we show that both K8 (G62C, K464N) and K18 (S230T) substitutions can severely affect the barrier function of a colonocyte monolayer *in vitro*. An important aspect of our findings is that these mutations were tested in the background of a pre-existent wild type K8/K18 filament network, as we compared mutant and wild type genetically engineered cell lines in the same genetic background of HT-29 colonocytes (isogenic cell lines). The data on K18 S230T are particularly interesting as it causes a high increase in K_d_ and a decrease in cell growth rate, despite a normal cell cycle profile. Exposure of K8/K18 mutants to heat-stress does not appear to induce much keratin aggregation and filament bundling as seen for mutant epidermal keratins, but instead causes the intestinal cell monolayers to detach from the surface as a sheet of cells. Tight junction proteins ZO-1 and claudin-4 are in the case of K18 S230T displaced, which has the potential to directly interfere with the epithelial barrier function. To further assess this data we developed a computer-generated model of the L12 linker region of the K8/K18 dimer. This was used for *in silico* testing of the impact the K18 S230T mutation has on the structure of this domain. Though the S230T substitution may not structurally alter the L12 domain in a major way, it may introduce a new hydrogen bond within the K18 chain, setting additional constraints to the flexibility of this region. We believe that K18 S230T is a mutation in its own right and similar to the mutations in K8 may rightfully predispose carriers to pathologies of the gastrointestinal tract by affecting the barrier function of the intestinal epithelium. Our data demonstrates for the first time that K8/K18 mutations may be able on their own to interfere with colonocyte function *in vitro*.

## Material and Methods

### Isogenic cell lines, design and cloning

HT-29 cells (HTB-38 from ATCC), a human colorectal adenocarcinoma cell line, were grown in culture at 37°C, 5% CO2, using DMEM (PAA, Austria) supplemented with 10% FBS (PAA, Austria), 1x antibiotic/antimycotic solution (Gibco, Life technologies, USA) and 1 x MEM non-essential amino acids (Gibco, Life technologies, USA), as culture medium. Cells were passaged at 70% confluence. K8 and K18 clones were generated as described previously [26]. In brief, site-directed mutagenesis was performed using the QuikChange kit (Stratagene, USA) to generate K8/K18 cDNA constructs with IBD patient mutations (K8 wild type (WT), K8 G62C, K8 K464N, K18 WT and K18 S230T). cDNA constructs were tagged at the N-terminus with a FLAG epitope and cloned into pcDNA3 vector (Invitrogen, Life technologies, USA). HT-29 cells were subsequently transfected using FuGene (Roche), according to manufacturers instructions. Wild type and mutant construct expressing cells were initially selected using 1 mg/ml of G418 (Gibco, Life technologies, USA). This was followed by two additional rounds of selection of stably transfected single clones in the presence of G418 at 0.5 mg/ml. To confirm that newly generated cell lines express the K8/K18 mutations above, we sequenced KRT8 and KRT18 from genomic DNA isolated from these cells, and assessed K8/K18 protein expression by immunoblotting. The following antibodies were used: anti-FLAG mouse monoclonal antibody M2 and anti-K8 mouse monoclonal antibody M20 (both from Sigma-Aldrich, USA), and anti-K18 mouse monoclonal antibody LDK-18 (gift from E.B. Lane). Mouse monoclonal antibody LJ4 was used to detect phosphorylated K8 (gift from B. Omary). The resulting cell lines were designated as HT-29+K8 WT (K8 WT control), HT-29+K8 G62C, HT-29+K8 K464N, HT-29+K18 WT (K18 WT control) and HT-29+K18 S230T, but for easier reading will be from now on referred to according to the construct they express.

### Permeability assay

In this assay 24-well cell culture inserts (ThinCert, Greiner BioOne, Germany) with a 0.4 µm pore size were used. Cells were grown on inserts for 2 weeks, at which point transepithelial membrane conductance (TEER) was measured to ensure that the colonocyte monolayer was confluent and tight junctions assembled. Lucifer yellow (LY, 444 Da), a small fluorescent molecule routinely used in permeability studies, was added at 0.1 mg/ml final concentration to the medium in the upper chamber of the ThinCert system. Cells were further incubated at 37°C for 2 hours, after which the medium in the bottom chamber was collected and its fluorescence measured on a spectrophotometer. The resulting data was used to calculate the permeability coefficient (Papp) of each cell line. All experiments were done in triplicate repeats and the standard deviation was calculated accordingly.

### Growth rates

K8 and K18 cell lines were plated at 20,000 cells per well on 24-well plates. Cells were grown in HT-29 growth media supplemented with 0.5 mg/ml of G418. After 1 week in culture cells were detached from the plates using trypsin and counted with an automated cell counter (Scepter cell counter, Millipore, USA). Experiments were done in triplicate repeats and the standard deviation was calculated accordingly.

### Heat-stress assay

Heat-stress assay was performed as previously described [27]. At regular time intervals (0 time point, 1 hour, 2 hours, 4 hours and 16 hours of recovery after stress) cells were fixed and stained using either the anti-K18 antibody LDK-18 or anti-K8 antibody M20 as primary, and fluorescently labelled goat anti-mouse (488 nm) as secondary antibody (Molecular Probes, Life technologies, USA). Images were acquired using a fully motorized Zeiss Axioplan 2 fluorescent microscope running of Axiovision software (Carl Zeiss, Germany).

### Immunolabelling of tight junction proteins

K8 WT and K18 S230T cells were grown on 13 mm coverslips over 2 weeks, after which they were fixed and stained according to manufacturer's instructions using as primary antibodies the anti-claudin-4 mouse monoclonal 3E2C1 antibody, and the N-terminus anti-ZO-1 polyclonal rabbit antibody (both from Invitrogen, Life technologies, USA). As secondary fluorescently labelled (488 nm) antibodies we used goat anti-mouse and goat anti-rabbit antibodies (Molecular Probes, Life technologies, USA).

### Total protein extracts

Cells were cultured as described above. After reaching 70–80% confluence cells were washed with PBS and lysed in extraction buffer containing: 50 mM Tris/HCl pH 7.5, 1 mM EDTA, 1 mM EGTA, 50 mM NaF, 5 mM sodium pyrophosphate, 10 mM β-phosphoglycerate and 1% Triton X-100, plus the following which were freshly added immediately prior to use: 1 mM Na-orthovanadate, 0.1% β-mercaptoethanol, 1 mM PMSF, 1 mM benzamidine, 10 µg/ml leupeptin and 10 µg/ml pepstatin A. Protein concentration was determined using the Bradford assay (Bio-Rad protein assay, Bio-Rad Gmbh, Germany) and 10 µg of total protein per sample was loaded per track on denaturing protein gels. Immunoblotting was carried out using monoclonal antibodies described above (against K8, K18, phospho-K8, ZO-1 and claudin-4).

### Calculation of dissociation constants (K_d_)

We re-analysed the previously published surface plasmon resonance data on K8 and K18 wild type and mutant recombinant proteins by Owens and colleagues [26] using ENZO: Enzyme Kinetics [28]. ENZO is a free web tool for building kinetic models of enzyme-catalysed reactions, evaluation of rival reaction schemes and routine tests in enzyme kinetics. ENZO allowed us to calculate the K_d_ of K8 and K18 mutants and compare them with wild type proteins (Table 1).

**Table 1:** K8/K18 mutations induce an increase in protein dissociation constants (K_d_).

Cell line	K_d_
K8 WT	8.5 nM
K8 K464N	13.4 nM
K8 G62C	26.2 nM
K18 WT	8.5 nM
K18 S230T	161 nM

The surface plasmon resonance data published by Owens and colleagues [26] was re-analysed using ENZO software (enzo.cmm.ki.si) to calculate the dissociation constants of the different recombinant proteins. The K_d_ of K18 S230T recombinant protein resulted to be the highest, suggesting a significantly lower affinity of K18 S230T for wild type K8 than in the case of K8 G62C and K8 K464N for wild type K18.

### Computer modelling of K8/K18 L12 region dimer

Modelling was performed using the crystal structures of vimentin (PDB code 3TRT) and Maf-G transcription factor (PDB code 3A5T) as the templates for the coil and linker regions, respectively [29], [30]. According to the alignment the residues were mutated with the Whatif molecular modelling tool. After structure optimization, a 1 ns constant pressure and temperature (CPT) dynamic simulation (300 K, 1 bar, time step 1 fs) invoking the EWALD summation for calculating the electrostatic interactions was run for each structure (wild type and the mutant). For averaging purposes the simulation was repeated four times in the case of wild type K8/K18 fragment and five times for the K8 WT/K18 S230T mutant. In all dynamic simulations 10240 water molecules were included. All molecular simulations and analyses were performed with CHARMM, a versatile molecular simulation program.

## Results

### Increased paracellular permeability of K8 and K18 mutant cell lines

**Figure 1:**
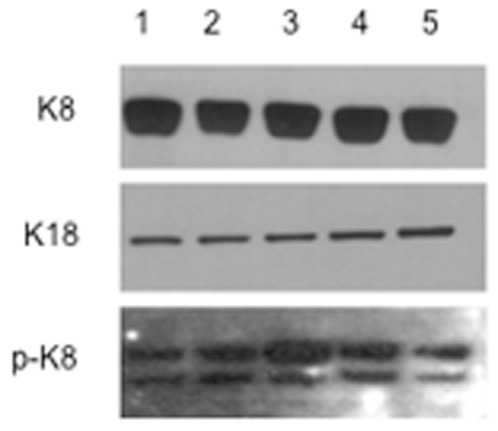
Western blot analysis of protein extracts from isogenic K8 and K18 cell lines. Tracks: (1) K8 WT cells; (2) K8 G62C cells; (3) K8 K464N cells; (4) K18 WT cells; (5) K18 S230T cells. Total protein extracts (10 µg) from selected clones were run on a 5% denaturing polyacrylamide gel, transferred to a PVDF membrane, and incubated with antibodies against K8 (M20), K18 (LDK-18) and phosphorylated keratin (LJ4) protein. As shown, K8 and K18 expression levels are very similar between the selected clones, while phosphorylated keratin levels are altered in the mutants, up-regulated in the K8 G62C (track 2) and K8 K464N (track 3) cells and down-regulated in the K18 S230T cells (track 5).

**Figure 2:**
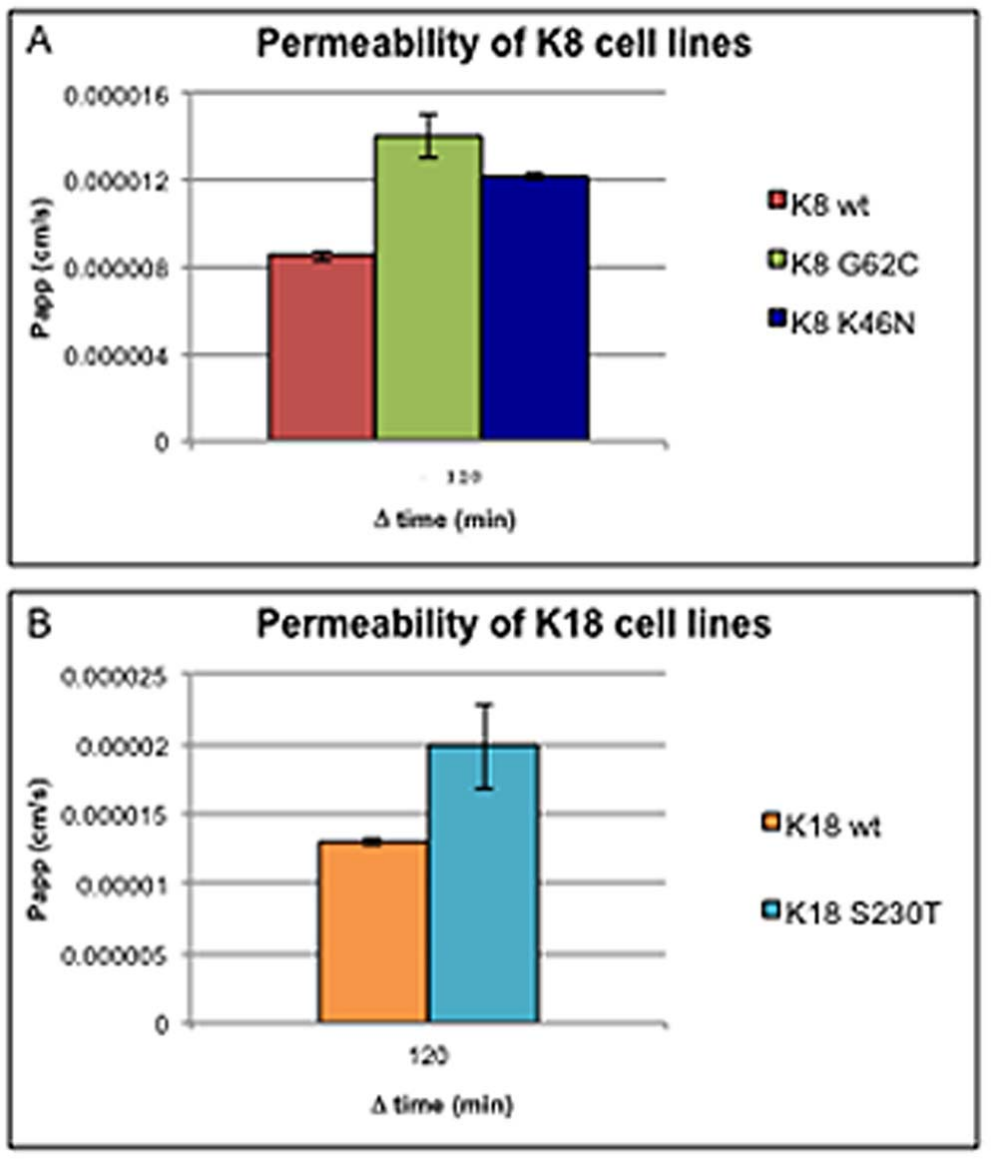
K8/K18 mutants have higher paracellular permeability. Isogenic K8/K18 cell lines were grown on cell culture inserts with a 0.4 µm pore size. Lucifer yellow was used to test the permeability of tight junctions after cells reached confluence and matured over a period of 2 weeks in culture. (A) Permeability coefficient (Papp) of K8 cell lines. Both mutants have a much higher permeability than the K8 control cell line. (B) Papp of K18 cell lines, where the K18 S230T mutant (similar to K8 mutants) displays a 30% higher permeability from the K18 control cell line.

Clones of HT-29 colonocytes stably transfected with K8 and K18 wild type and mutant constructs (K8 WT, K8 G62C, K8 K464N, K18 WT and K18 S230T) were engineered and selected according to the methodology described in Materials and Methods. Clones with similar levels of K8 and K18 expression were selected for further experiments (Fig.1). As shown, no major differences in K8 and K18 protein levels could be observed between the clones selected. However, the level of keratin phosphorylation appears somewhat altered in two of the three mutants analysed (Fig.1), i.e. increased in the K8 G62C and K8 K464N clones in comparison to their K8 WT control cells, and decreased in K18 S230T in comparison to K18 WT control cells. Cells were grown on cell culture inserts commonly used for permeability studies. The “leakiness” of confluent cell monolayers was measured by following the diffusion rate of lucifer yellow (LY), a small fluorescent molecule. As small molecules are more likely to cross sheets of epithelial cells by simple diffusion than by a receptor or energy dependent mechanism, any detected LY in the basal compartment of this culture system is due to the paracellular transport mechanism. The tighter tight junctions lock colonocytes together into a monolayer the lower is the permeability of the epithelial sheet, and vice-versa. The results of the permeability assays are presented in Fig. 2. As shown, in vitro cell monolayers of the two K8 mutants (K8 G62C and K8 K464N, Fig. 2A) have a significantly higher permeability coefficient (Papp) from the K8 WT control cell line. Surprisingly, a similar result was also obtained for colonocytes expressing the K18 S230T mutation, which have a much higher permeability for LY than K18 WT control cells (Fig. 2B).

### Distribution of tight junction proteins ZO-1 and claudin-4 is altered in K18 S230T cells

**Figure 3:**
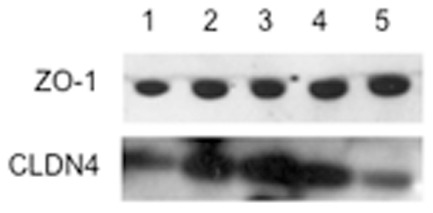
Western blot analysis of tight junction components in protein extracts from isogenic K8 and K18 cell lines. Tracks: (1) K8 WT cells; (2) K8 G62C cells; (3) K8 K464N cells; (4) K18 WT cells; (5) K18 S230T cells. Total protein extracts (10 µg) from selected clones were run on a 4–12% denaturing polyacrylamide gel, transferred to a PVDF membrane, and incubated with antibodies against ZO-1 (N-term) and claudin-4 (3E2C1). While ZO-1 expression levels are similar, claudin-4 levels are elevated in the two K8 mutants (tracks 2 and 3) and down-regulated in the K18 S230T cells (track 5).

**Figure 4:**
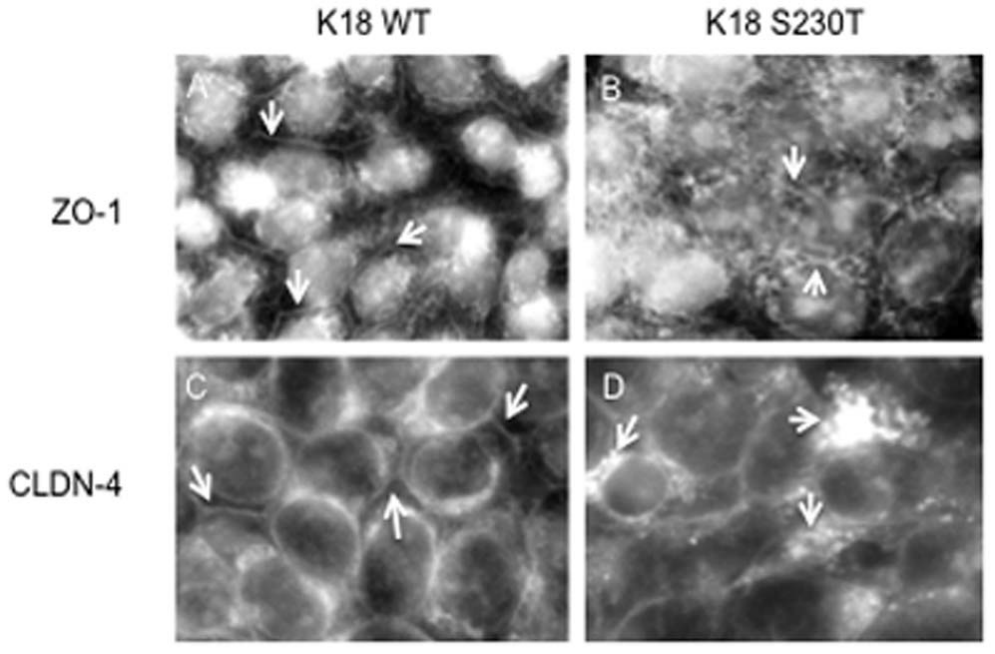
The distribution of ZO-1 and claudin-4 is altered in the K18 S230T mutant. Colonocytes stably expressing the K18 S230T mutation were immunofluorescently labelled for ZO-1 (upper panels, A and B) and claudin-4 (bottom panels, C and D), important components of tight junctions. In both cases the K18 control cells (panels A and C) show a more regular pattern of staining that traces the cell membrane. In contrast, the K18 S230T mutant (panels B and D) has a very diffuse pattern of staining for ZO-1 and claudin-4, and smaller protein aggregates are also visible (see arrows). Images were acquired with a 60x objective lens.

The increased permeability rate of the K8/K18 mutants prompted us to check the protein levels of certain components of tight junctions, namely ZO-1 and claudin-4 (Fig. 3). While protein levels of ZO-1 appear similar between these cell lines, the levels of claudin-4 differ substantially. As shown, the K8 G62C and K8 K464N mutants display an increase in claudin-4 in comparison to K8 WT, while in the K18 S230T mutant this appears decreased in comparison to the K18 WT control. We found the data on K18 S230T particularly interesting and so also examined the distribution of ZO-1 and claudin-4 in these cells by immunofluorescent labelling (Fig. 4). Figure 4A, B are the K18 WT and K18 S230T cells respectively, stained with an antibody against ZO-1, while in Figure 4C, D are the same cells stained with an antibody against claudin-4. In both cases the staining is more regular in K18 WT overexpressing cells, where it traces the cell membrane as expected for tight junctions (see arrows). In contrast, ZO-1 and claudin-4 seem to have a diffuse distribution in K18 S230T mutants, with visible speckles and aggregates (arrows). Hence, tight junctions of an epithelial sheet consisting of colonocytes expressing the K18 S230T mutation may have a compromised function, just as previously indicated by the permeability assay.

### K18 S230T colonocytes have low growth rates

**Figure 5:**
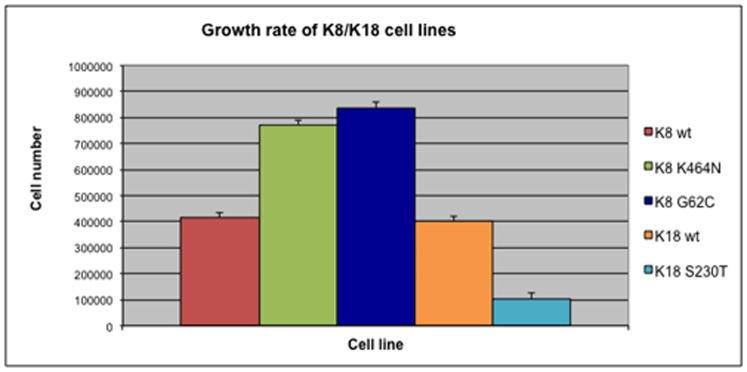
K8/K18 mutants have altered growth rates. Cells were grown on 24-well cell culture plates. After a week in culture cells were trypsinized and counted. As shown, the K8 mutants grow at a much higher rate than the K8 WT cell line, while the K18 S230T cells grow very slow, having only a quarter of the growth rate of K18 WT cells. All cell lines (mutant and wild type) have a normal cell cycle profile (not shown).

Next we decided to test whether these mutations interfere with cell growth. Although all cell lines appear to have normal cell cycle profiles (based on flow cytometry with propidium iodide staining, data not shown), their growth rates differ significantly. The results of these assays are summarized in Fig. 5. Both control cell lines (K8 WT and K18 WT) display similar growth rates, suggesting that the extra copy of the wild type gene on its own does not interfere with growth. The two K8 mutants (K8 G62C and K8 K464N) grow considerably faster, with a growth rate that is almost the double of the wild type. On the contrary, the K18 S230T cells differ greatly as they grow much slower than the K18 WT control cells.

### Heat-stress causes K8/K18 mutants to detach from the surface as a sheet of cells

**Figure 6:**
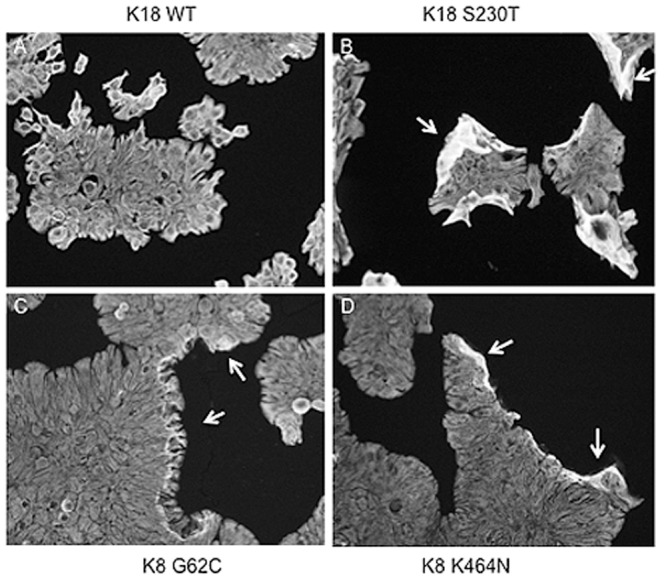
Effect of heat-stress on K8/K18 mutants. Cells were grown on coverslips until they formed smaller patches of cells, after which they were exposed to heat-stress for a brief period of time. At 0 time, 1 hour, 2 hours, 4 hours and 16 hours of recovery, cells were fixed and immmunofluorescently labelled for either K8 or K18 protein. Images shown are representative for the 1 hour time point of recovery, when the biggest differences were observed. In panel A are the K18 wild type control cells and these appear unaffected by heat stress. Panels B, C and D are the K18 S230T, K8 G62C and K8 K464N mutants respectively. Arrows mark border regions where cell sheets have detached from the surface and curled up against it. Images were acquired with a 20x objective lens.

As the effect of the K18 S230T mutation appears to act more severely on cell function than previously concluded based on recombinant IF protein polymerization experiments [26], we decided to challenge these cells with heat-stress. The heat-stress assay proved useful in testing the IF network resilience for epidermal keratins. Cells were grown on coverslips for several days to allow them to attach firmly and form larger groups of cells. Cells were then subjected to heat stress and left to recover at normal growth temperature. At regular time intervals coverslips were fixed and immunofluorescently labelled to visualize the keratin cytoskeleton. The greatest differences between wild type and mutant cells were observed at 1 hour of recovery after heat-stress. Figure 6A, B are representative images of K18 WT and K18 S230T cells, while Figure 6C, D are representative images for K8 G62C and K8 K464N cells respectively. As shown, at this stage the free edges of islands of K8/K18 mutant cells (see arrows) appear much thicker in comparison to wild type cells. This is due to the detachment of border cells from the surface whilst remaining attached one another, causing the monolayers to curl up against the sheet of attached cells.

### The K_d_ of K18 S230T is significantly higher than of K8 mutants

As the dissociation constant (K_d_) is used in chemistry as measure of affinity between two molecules, we re-analysed surface plasmon resonance data previously published [26]. The web tool ENZO allowed us to calculate the K_d_ of K8 and K18 wild type and mutant proteins (Table 1). The higher the K_d_, the lower the affinity between two molecules. Although all mutations appear to decrease the affinity of the mutant protein to their respective wild type partner, the data on K18 S230T is striking, having the highest K_d_ among these mutations.

### Molecular modelling of the K8/K18 L12 dimer region

**Figure 7:**
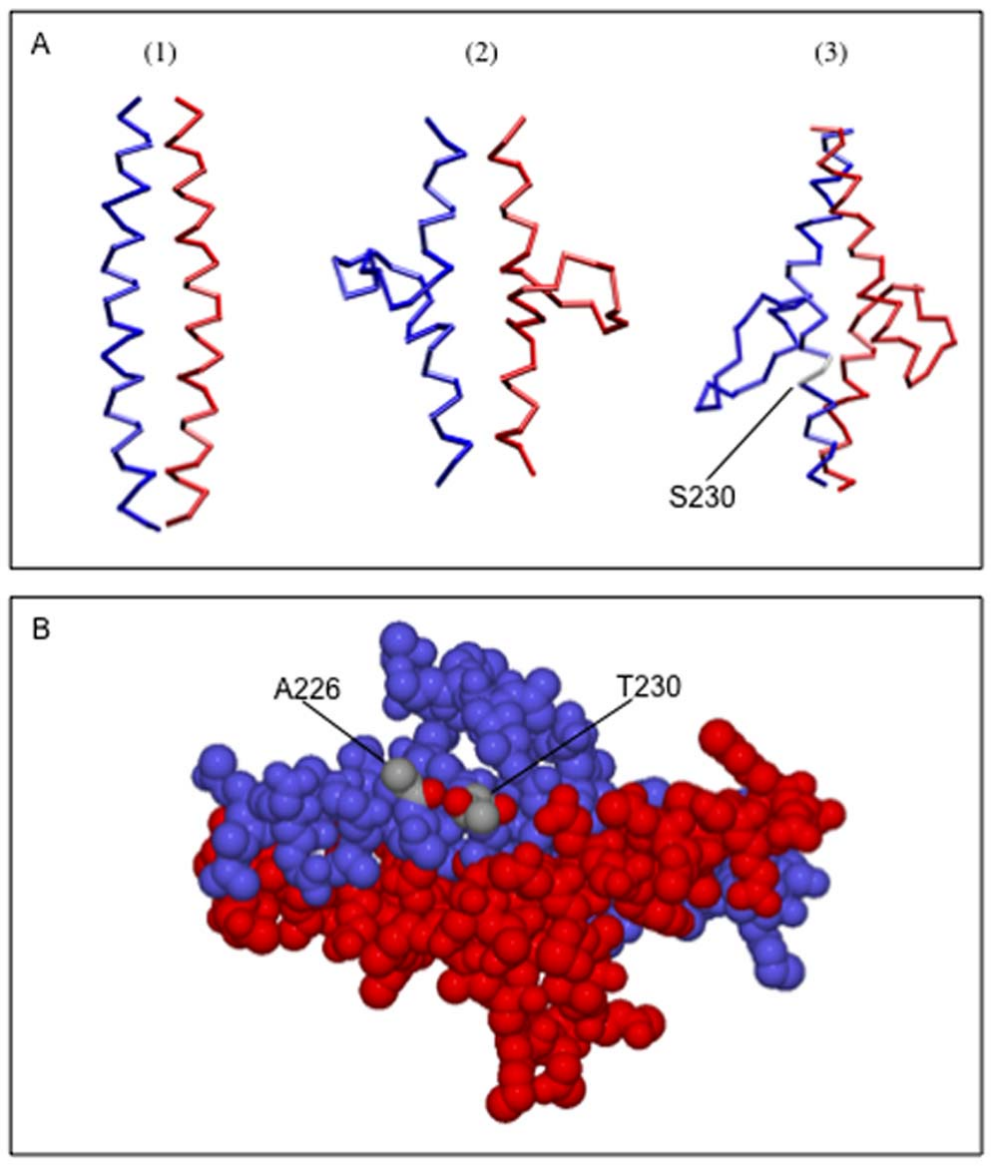
Molecular modelling of the K8/K18 L12 domain. We used 134 residues from the K8 and K18 polypeptides, which span the end part of helix 2A, the L12 linker and the beginning of helix 1B. The helical regions in our model were built based on the crystal structure of vimentin (PDB code 3TRT), while the linker domain on Maf-G protein (PDB code 3A5T). (A) The three possible models of the L12 region of K8/K18 protein folding. Models 1 and 2 were discarded, as they do not take into account many of the known keratin heterodimer structural features. Model 3 was further refined as the K8/K18 chains form in the L12 linker region an interchanging loop with a short four-helix bundle. The position of the S230 residue of K18 is labelled white. (B) In case of the S230T substitution the threonine residue cannot rotate freely as serine due to its size. Also, a new hydrogen bond may form between residues T230 and A226 of K18, thus stabilizing this part of the protein.

**Figure 8:**
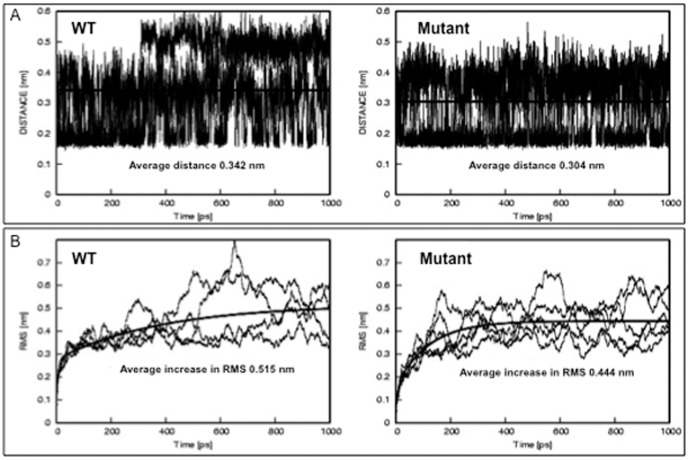
K18 S230T may form an additional hydrogen bond within the K18 chain in the L12 linker. Molecular dynamics experiments were performed on the K8/K18 L12 linker model with the duration of 1 ns. This was repeated 4 times for the wild type sequence and 5 times for the mutant. The resulting data was used to calculate: (A) the average distances between the hydroxyl group of SER/THR230 and the backbone oxygen of ALA226 in K18, which in the case of the mutant (THR230) falls within the range of a moderately strong hydrogen bond (2.5–3.2 Å); (B) the relative root mean square (RMS) along the run, a measure of dimer stability. Both parameters indicate that the K18 S230T mutation may be forming an additional hydrogen bond within the K18 chain, which would be expected to increase the rigidity of this part of protein to additional conformational pressures.

To test the effect of the K18 S230T mutation on the L12 region of the K8/K18 keratin heterodimer, we generated a 3D model of the L12 linker along with several adjacent residues of the 2A and 1B helices (from amino acid 181 to 315 of both K8 and K18, in total 134 residues of each chain). The linker domain was modelled according to the crystal structure of Maf-G transcription factor (PDB code 3A5T), while the helices according to vimentin (PDB code 3TRT). Since the crystal structure of any keratin protein is still unknown, we identified three possible L12 linker models, which are based on the following concepts (Figure 7A): (1) The linker region is just a linear continuation of the coiled-coil, disrupting the coiled-coil in a similar fashion like the stutter region of coil 2B; (2) The linker region is a bubble between coils 2A and 1B, allowing the main chain to conserve the characteristic seven amino acid repeat (this would be the case if the entire protein had only one origin of protein folding; (3) The two keratin chains form an interchanging loop with a short four helix bundle in the L12 linker region (this is the case of a protein having several origins of protein folding). In the resulting structures the K18 S230T mutation is positioned at the beginning of the linker region. Models (1) and (2) were discarded. Model 1 seemed unlikely as the 23 amino acid long (the L12 region) disruption of the heptad repeat within the helical domains would introduce great instability into the K8/K18 heterodimer coiled-coil. On the other hand model 2 is not realistic as it predicts only one origin of protein folding, and keratin monomers are between 400 and 600 amino acids long. Therefore such a simplified model would also disregard any possible influence of the loop region to the coiled-coil structure of the K8/K18 heterodimer. However in model 3 the K18 S230T mutation lies at the beginning of the four-helix bundle region, which can only form if the keratin monomer has several origins of folding, and it also suggests having impact on the structure's dynamics. We performed molecular dynamics on model 3, which indicated the possibility that the K18 S230T mutation introduces an additional hydrogen bond into the structure (Fig. 7B). From the molecular dynamics data we calculated two key parameters: the average distance between the hydroxyl group of SER/THR230 and the backbone oxygen of ALA226 in K18 (Figure 8A), and the relative root-mean-square along the run, which is a measure of dimer stability (Fig. 8B). Both parameters suggested an additional hydrogen bond in the T230 mutant, stabilizing the structure and increasing the rigidity of the mutant L12 linker region.

## Discussion

We have here shown that not only some K8 variations, but also the disputed K18 S230T change, are able to produce a phenotype in an *in vitro* intestinal epithelium model system. By introducing the K8 (G62C and K464N) and K18 (S230T) mutations into the background of the same wild type HT-29 colonocyte cell line, we created isogenic cell lines and eliminated potential variations arising from different genetic backgrounds. We observed increased paracellular permeability in all three mutants compared to their corresponding isogenic wild type control cells (HT-29 cells with an extra copy of the K8 or K18 wild type genes). Unlike mutant epidermal keratins, where heat-stress generally induces keratin aggregation and filament bundling, the K8/K18 mutations we tested appear to affect cell adhesion and perturb tight junctions. The paracellular route of transport is primarily dependent on tight junctions. We already demonstrated that severe K5/K14 mutations (giving rise to a skin fragility disorder in humans) affect the expression and distribution of cell junction proteins, principally desmosomal components [19]. Like desmosomes, tight junctions are also multiprotein intercellular adhesion complexes with a defined stoichiometry, which is necessary to ensure their primary function: locking epithelial cells tightly together into a selective barrier that protects underlying tissues from pathogens and toxins. As we were particularly interested in the K18 S230T mutant, we tested by Western blotting and immunofluorescence if tight junction proteins were affected. We found that proteins ZO-1 and claudin-4 have a diffuse distribution in the K18 S230T mutant, and that claudin-4 is down-regulated in these cells, suggesting impaired function of tight junctions. Therefore, under in vitro conditions mutant keratins K8/K18 appear able to affect two major roles of intestinal epithelia: tissue permeability and tissue fragility. The mechanism how these mutations interfere with cell function is unclear, however it seems to be linked to cell junction complexes. The keratin IF network assembly, dynamics and maintenance are dependent on the interaction of the keratin network and precursors with both microtubules and actin filaments [6], [31]. As the three cytoskeletal filament systems interact between them and bind to cell junction proteins, changes in one filament system may be able to interfere with the function and/or distribution of distant proteins through their binding partners [32], [33]. An interesting aspect of this is that the K8 and K18 mutant cell lines analysed here do show some differences in keratin phosphorylation status, which is reflected by alterations in protein levels of claudin-4.

Evidence of a link between simple epithelial keratins and IBD has been previously shown on animal models. K8 null mice develop colitis, rectal prolapse and hyperplasia of the colon. Histological analysis suggests that the colonic inflammation seen in K8 null mice might be the result of an epithelial rather than immune system defect [34]. In addition, K8 null mouse colonocytes display a resistance to apoptotic stimuli, which is considered a protective function [35]. Both inflammation and resistance to apoptosis was treatable with antibiotics, suggesting the primary defect lies in the intestinal epithelium, while inflammation is the result of a subsequent immune response to luminal bacteria. It has also been shown that colons of K8 null mice have altered electrolyte transport across the barrier caused by ion transporter mistargeting, which probably accounts for the diarrhoea in these animals [36], [37]. However studies focused on the genetic analysis of genomic DNA of groups of patients with IBD failed to show an explicit link between K8/K18 mutations and IBD development, at least not in the way epidermal keratins are linked to some skin disorders [27], [38], [39]. Firstly, the percentage of IBD patients carrying a K8/K18 mutation is low, somewhere between 2–10% of the total number of patients involved in the study. Secondly, in contrast to epidermal keratins, where mutations predominantly affect the helical domains of the protein and physically perturb the IF network, the K8/K18/K19 mutations found in IBD patients lie in the non-helical end domains, generally expected to produce a milder phenotype. It was therefore presumed that mutations in simple epithelial keratins act indirectly and in concert with some other susceptibility factors, making carriers susceptible to IBD development.

In 2004, Owens and colleagues [26] identified the first K8 mutations in IBD patients (G62C, I63V and K464N). These are situated in the end domains of K8 and accounted for only 5% of the patients included in the study. A K18 mutation (S230T) was also found within the L12 linker domain, which in epidermal keratins is associated with milder disease phenotypes. Experiments (surface plasmon resonance, sedimentation assay, *in vitro* filament assembly, de novo filament polymerization) with purified recombinant wild type and mutant K8/K18 protein led to the conclusion that K8 mutations may interfere with keratin filament assembly. This was in particular true for the G62C mutation, which is now also associated with cryptogenic cirrhosis, pancreatitis, liver disease and primary biliary cirrhosis. On the other hand, although the K18 S230T variation was found in surface plasmon resonance experiments to interfere with the binding of monomeric K18 S230T to immobilized wild type K8, it was the only change to affect a non-conserved residue, not to affect *in vitro* filament assembly and was also found in four individuals from the control group. Consequently it was designated as a polymorphism. By using ENZO: Enzyme Kinetics software [28] we now calculated the dissociation constants (K_d_) from the published surface plasmon resonance graphs [26]. The highest K_d_ and therefore the lowest affinity for the immobilized keratin partner was determined for the K18 S230T protein, followed by K8 G62C and K8 K464N. Nevertheless it has been shown that once bound these K8/K18 heterodimer complexes are relatively stable [26].

In this study we also developed the first computer-generated model of the keratin L12 linker region and analysed the potential effects of K18 S230T *in silico*. Our model suggests that T230 establishes a new hydrogen bond with residue A226 of the same K18 chain. In theory the resulting stabilization of the protein could slow down protein turnover and thus affect the rate of cell growth. Interestingly, our K18 S230T colonocytes do have a lower growth rate than control cells.

To conclude, this is the first time intestinal cells expressing K8 or K18 mutations have been proposed as directly functionally impaired by these. We have shown that although in the background of endogenous wild type keratin protein, certain K8 and K18 mutations may be able to affect the permeability of intestinal epithelium and its resilience to stress. Although our data is based on an *in vitro* colonocyte model system, which may not exactly reproduce the situation found in the epithelial lining of the intestine *in vivo*, this still suggests that K8 and K18 mutations may be able to produce a phenotype on their own. The combined action of these with the immune response may certainly induce a variety of symptoms frequently found in patients with IBD.
