# Correction: Intestinal Cell Barrier Function *In Vitro* Is Severely Compromised by Keratin 8 and 18 Mutations Identified in Patients with Inflammatory Bowel Disease

**DOI:** 10.1371/journal.pone.0104122

**Published:** 2014-07-31

**Authors:** The PLOS ONE Staff

The images for Figures 2, 5 and 8 appear at a low resolution. Please see the high resolution images for Figures 2, 5 and 8 here.

**Figure 2:**
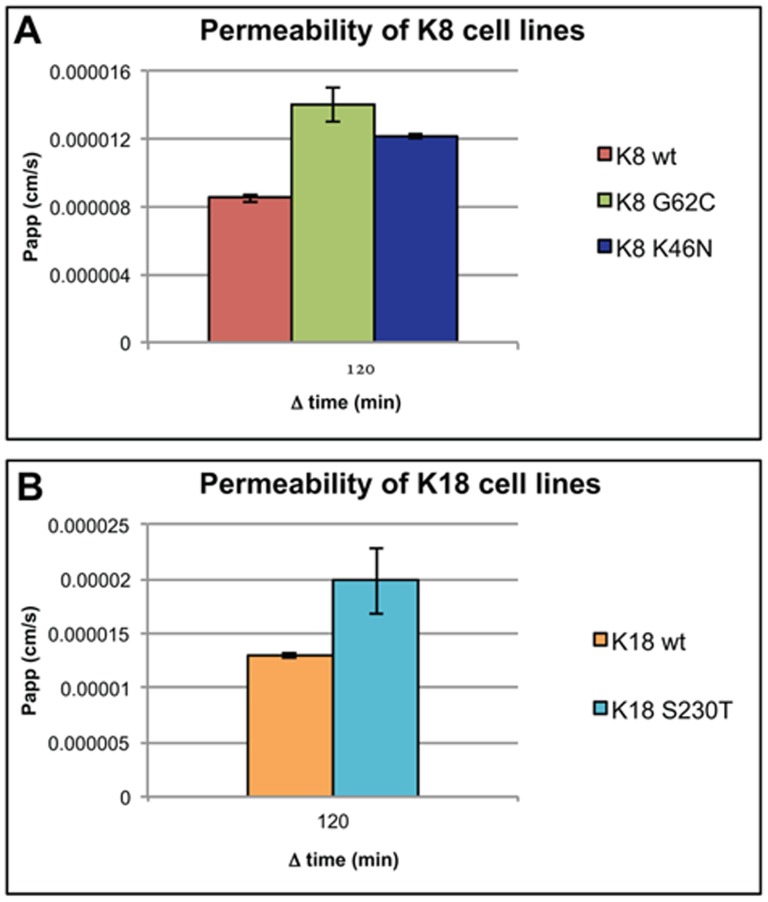
K8/K18 mutants have higher paracellular permeability. Isogenic K8/K18 cell lines were grown on cell culture inserts with a 0.4 µm pore size. Lucifer yellow was used to test the permeability of tight junctions after cells reached confluence and matured over a period of 2 weeks in culture. (A) Permeability coefficient (Papp) of K8 cell lines. Both mutants have a much higher permeability than the K8 control cell line. (B) Papp of K18 cell lines, where the K18 S230T mutant (similar to K8 mutants) displays a 30% higher permeability from the K18 control cell line.

**Figure 5:**
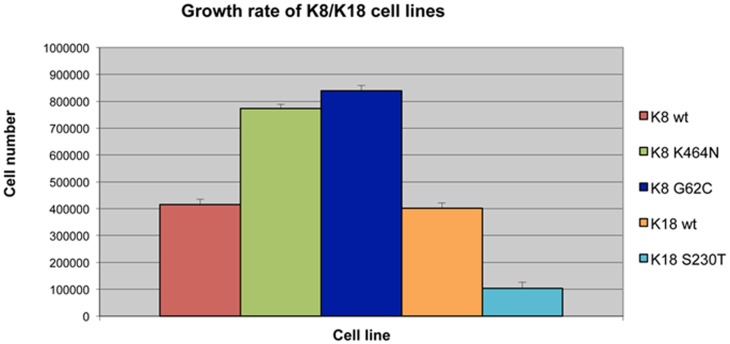
K8/K18 mutants have altered growth rates. Cells were grown on 24-well cell culture plates. After a week in culture cells were trypsinized and counted. As shown, the K8 mutants grow at a much higher rate than the K8 WT cell line, while the K18 S230T cells grow very slow, having only a quarter of the growth rate of K18 WT cells. All cell lines (mutant and wild type) have a normal cell cycle profile (not shown).

**Figure 8:**
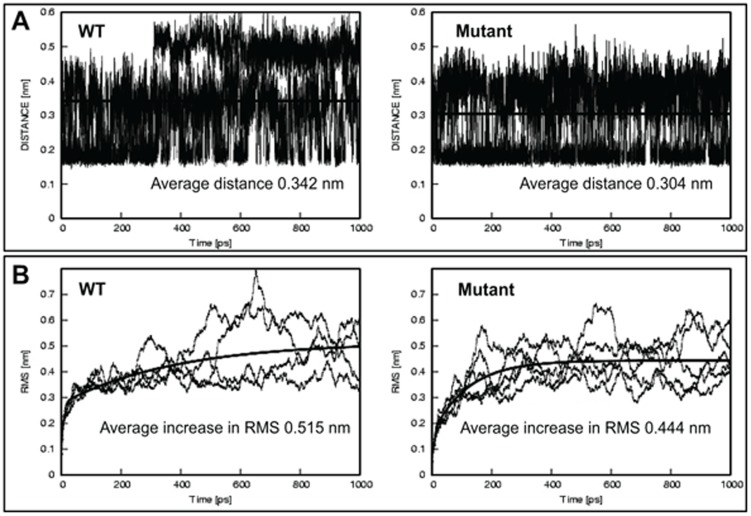
K18 S230T may form an additional hydrogen bond within the K18 chain in the L12 linker. Molecular dynamics experiments were performed on the K8/K18 L12 linker model with the duration of 1 ns. This was repeated 4 times for the wild type sequence and 5 times for the mutant. The resulting data was used to calculate: (A) the average distances between the hydroxyl group of SER/THR230 and the backbone oxygen of ALA226 in K18, which in the case of the mutant (THR230) falls within the range of a moderately strong hydrogen bond (2.5–3.2 Å); (B) the relative root mean square (RMS) along the run, a measure of dimer stability. Both parameters indicate that the K18 S230T mutation may be forming an additional hydrogen bond within the K18 chain, which would be expected to increase the rigidity of this part of protein to additional conformational pressures.
